# Affiliation as a Social Tension Buffer in the Aftermath of Street Fights

**DOI:** 10.1007/s12110-026-09516-1

**Published:** 2026-03-17

**Authors:** Laura Pighini, Ivan Norscia, Marie Rosenkrantz Lindegaard, Virginia Pallante

**Affiliations:** 1 Netherlands Institute for the Study of Crime and Law Enforcement (NSCR), Amsterdam, The Netherlands; 2 Department of Sociology, VU University Amsterdam, Amsterdam, The Netherlands; 3 Department of Life Sciences and Systems Biology, University of Turin, Turin, Italy

**Keywords:** Conflict management, Third parties, Interaction ethology, Video analysis

## Abstract

Naturalistic observations of conflict events documented that, similarly to nonhuman primates, human bystanders take an active role in managing the negative consequences of the conflict. However, although in nonhuman primates it has been suggested that bystanders affiliate with each other to decrease the social tension generated by the conflict, in humans affiliation as a stress-response in naturally occurring conflicts has not been documented yet. By applying observational methods from primatology, we conducted video analysis of streets fights recorded by surveillance cameras in public spaces of Amsterdam in order to evaluate whether conflicts promote bystanders’ affiliation as a tension-reduction mechanism. We found that witnessing a street fight increased the expression of anxiety-related behaviours in bystanders and their propensity to engage in non-physical and physical affiliative interactions with other bystanders and the conflict parties. The occurrence of physical affiliation improves in turn the emotional state of bystanders by reducing the expression of anxiety-related behaviours. By offering a behavioural-based analysis of the role of social affiliation in the post-conflict context, our approach allows to reveal that some mechanisms to cope with social tension observed in nonhuman primates can lead to similar management dynamics in human bystanders.

**Supplementary Information:**

The online version contains supplementary material available at 10.1007/s12110-026-09516-1.

## Introduction

In social animal species like human and non-human primates, conflicts are often social matters (de Waal, 2000). Bystanders who assist to a conflict can become part of its dynamics and play a key role in mitigating its negative effects, thus representing a potential resource for conflict management (Aureli & de Waal, 2000; Philpot et al., 2020). Therefore, understanding what factors affect bystanders’ propensity to intervene is key to uncover the mechanisms for bystanders’ action. However, while nonhuman primates’ literature offers extensive evidence on bystanders’ behaviour in naturalistic settings, from lemurs to apes (e.g., Aureli & de Waal, 2000; Brooker et al., 2024; Norscia & Palagi, 2016), ethological studies on humans spontaneously interacting in outdoor settings are still limited.

In humans, the study of bystanders’ behaviour primarily took place in laboratory settings, with a focus on bystanders as “inactive” agents and on the factors inhibiting their actions (Darley & Latané, 1968). However, a growing number of real-life video observational studies recently offered valid insights into the behaviour of bystanders in naturally occurring conflicts, revealing, in contrast, their active role. These new findings highlighted the importance of understanding the determinants of bystander activity. First, bystanders can become involved at different stages in a conflict. They can intervene by using physical force to de-escalate the conflict and to separate the conflict parties (Ejbye-Ernst et al., 2020, 2022), or they can engage in consolation behaviour in the aftermath of the conflict by offering comfort and help to victims (Liebst et al., 2024; Lindegaard et al., 2017). Second, social relationships between bystanders and victims were a key predictor of bystanders’ willingness to intervene (Liebst et al., 2019). The danger level in the conflict also mattered for whether and when bystanders would intervene in conflicts on the street (Lindegaard et al., 2022).

Ethological studies conducted on children suggest that an additional key predictor for intervention is the emotional state of bystanders. In pre-school children, conflicts generate social tension, with antagonists showing higher levels of anxiety-related behaviours in the post-conflict context (Fujisawa et al., 2006). The occurrence of bystander intervention as a response to a stressed victim suggests that this type of affiliation is driven by the perceived – and possibly shared – emotional state, as predicted by several empathy models (e.g. Russian-Doll Model by de Waal & Preston, 2017; Neurocognitive model of emotional contagion by Prochazkova & Kret, 2017; prosocial behaviour based on empathy by Decety et al., 2016). The occurrence of affiliation has in turn a calming effect on the receiver, reducing their anxiety level (Fujisawa et al., 2006). Ethological observations in conflict situations occurring in public spaces documented an increase of social tension also in human adults (Pallante et al., 2023, 2024). In this context, it was observed that people engaging in street fights express a state of anxiety through specific behavioural markers (e.g., displacement activities) – in part overlapping with those observed in nonhuman primates – which increase in the aftermath of the conflict compared to a baseline situation (Pallante et al., 2024). The emotional state of bystanders, however, has been considered only in experimental research. Research on human adults conducted in laboratories settings documented that bystanders show an increase of stress when witnessing a conflict, which has a negative effect on bystanders’ motivation to help (Hortensius & de Gelder, 2018; Hortensius et al., 2018). However, the possible role of social tension in affecting bystanders’ dynamics in conflicts remains speculative, as empirical evidence in real-life events is currently lacking.

The observational studies conducted on human conflicts are based on a theoretical framework developed in ethological research. In nonhuman primates’ literature, several models have been developed to explain bystander intervention. What these models have in common, is that they predict that bystander activity in conflict events is affected by the social tension that the conflict generates (Decety et al., 2016; Preston & de Waal, 2002; Prochazkova & Kret, 2017; Yamamoto, 2017). Experiencing social tension produces in turn a twofold effect on bystander behaviour. It can promote empathy-driven behaviours towards the victim of a conflict (Palagi & Norscia, 2013; Palagi et al., 2014; Romero et al., 2010) and it can facilitate bystander-bystander affiliation as a tension-reduction mechanism (Daniel & Alves, 2015; De Marco et al., 2010; Judge & Mullen, 2005).

In several nonhuman primate species, it has been documented that after being involved in a conflict, victims increase their anxiety level (Clay & de Waal, 2013; Fraser et al., 2008; Palagi et al., 2014). This emotional state can be transferred from the victim to the bystanders, driving their intervention. The spontaneous post-conflict affiliation offered by the bystanders third parties not involved in the conflict to the victim (a form of triadic contact, sensu Castles, 2000) is defined as consolation when it works in alleviating the stress in the victim (de Waal, 2008; Fraser et al., 2008). However, consolatory interventions might reduce distress not just in the victim, but also in the consoler (Clay & de Waal, 2013; de Waal & Preston, 2017; Fraser et al., 2008; Romero et al., 2010). Similar to victims, also bystanders who witness a conflict increase their anxiety level (Cordoni et al., 2025; Daniel & Alves, 2015; De Marco et al., 2010; Judge & Mullen, 2005; Majolo et al., 2009; Schino & Sciarretta, 2015). To cope with that, bystanders can affiliate with each other (quadratic interactions), a type of interaction that literature on nonhuman primates reports as having a function in reducing social tension (Cordoni et al., 2025; Daniel & Alves, 2015; De Marco et al., 2010; Judge & Mullen, 2005). As a whole, in nonhuman primates a state of anxiety arising from the witnessing of a fight or from an empathic process is, therefore, associated with an increased sociality of bystanders, which ultimately improves bystander’s emotional state.

Taken together, these pieces of evidence raise questions on the role of anxiety in regulating human bystanders’ response in conflict events. To fill this gap, this study aims at analysing video recorded street fights to understand the occurrence of bystanders’ affiliation as a tension-reduction mechanism. We proceeded as following:

First, we evaluated whether bystanders increase the expression of anxiety after the occurrence of a conflict. From an ethological standpoint, anxiety – a proxy for stress – in primates (including humans) can be reliably measured through the observation of displacement behaviours (e.g., scratching and self-grooming; Maestripieri et al., 1992), that can be expressed by the subjects after a stressful event, such as a conflict. In nonhuman primates, the occurrence of a conflict increases social tension in the group leading to higher rates of displacement behaviours in bystanders (Cordoni et al., 2025; Daniel & Alves, 2015; De Marco et al., 2010; Judge & Mullen, 2005). The expression of anxiety through displacement behaviours because of a stressful event have been observed in children and adults who were involved in aggressive interactions, showing that human and nonhuman primates express anxiety through similar behavioural indicators (e.g., self-touching, scratching; Fujisawa et al., 2005; Maestripieri et al., 1992; Pallante et al., 2023; Troisi, 2002). Therefore, we expected an increase of anxiety-related behaviours in bystanders when witnessing a street fight.

Second, we evaluated whether the occurrence of a conflict leads to an increase in affiliative interactions of bystanders, both with other bystanders and the antagonists. Along with the increase of anxiety, observations of nonhuman primates also report an increase in affiliative behaviour of bystanders, who tend to approach the conflict parties and the other bystanders (De Marco et al., 2010; Judge & Mullen, 2005). Affiliative coping under stress has been documented also in humans. Research conducted in laboratory settings showed that acute stress can increase the propensity to affiliate and to prosocially interact. Studies on “tend-and-befriend” responses (Taylor et al., 2000) and social buffering mechanisms (e.g., Heinrichs et al., 2003; Kirschbaum et al., 1995) reported that social contacts often serve a coping or tension-reduction function under stress. Similarly, exposure to distress or conflict can increase empathy and altruistic help (Buchanan & Preston, 2014; de Waal & Preston, 2017; von Dawans et al., 2012). However, few naturalistic, behaviourally grounded studies based on public conflict events show a general tendency of bystanders to affiliate (Au-Yeung et al., 2023; Lindegaard et al., 2017; Liebst et al., 2024). Both contact and non-contact (vocal) affiliation may increase after a conflict in humans (Fujisawa et al., 2006; Liebst et al., 2024; Lindegaard et al., 2017) and in nonhuman primates (Cheney & Seyfarth, 2018; Gros-Louis et al., 2008). Thus, we expected that contact and non-contact affiliation would increase in bystanders when witnessing a street fight. Since long-lasting contacts might have a different meaning from short contacts and be more effective in calming the receiver (Aureli & Schino, 2022), we made a distinction between short affiliative contacts and long affiliative contacts.

Finally, we investigated whether bystander affiliations (with an antagonist or another bystander) could increase the expression of anxiety in bystanders. The buffering effect of affiliation has been documented in the post-conflict context in nonhuman primate bystanders, where the anxiety-related behaviours expressed after a conflict decreased after affiliation (Daniel & Alves, 2015; Judge & Mullen, 2005; Schino & Sciarretta, 2015). Although to our knowledge, no information is available for human bystanders in real-life conflicts, it has been reported that affiliation improves the emotional state of the people who experienced a stressful stimulus (Grandi, 2016; Kikusui et al., 2006; Morrison, 2016). Therefore, we expected that affiliative behaviours would be followed by a decrease of anxiety-related behaviours in the bystanders involved in affiliative behaviours with other bystanders or with the antagonists of the conflict.

## Materials and Methods

### Video Sample and Subjects

We analysed CCTV footage interpersonal conflicts recorded by the police of Amsterdam (The Netherlands) through surveillance cameras installed in public spaces of Amsterdam during the year 2017, 2020 and 2023. Our access to the footage was provided under strict data storage and processing conditions, similar to other video-observational studies (see Nassauer & Legewie, 2022).

The CCTV cameras were located in different neighbourhoods of the cities, including the inner entertainment areas, central business districts and suburban locations. We instructed camera operators to record the occurrence of conflict events, from quarrels indicated by aggressive gesturing and frustration to serious violence involving hitting and kicking someone on the ground. The visual range covered by the cameras varied in size. It could extend from narrow streets of the city centre, about 5 m wide, to large squares, which could measure up to 200 m x 100 m. The cameras were equipped with a pan-tilt zoom, which allowed the camera operators to zoom in on an event and move to follow a subject that was moving out of the frame. Each clip had a variable duration spanning from few minutes to approximately one hour. The footage did not include sound.

From a total sample of 500 recorded incidents from the year 2017, 1000 recorded incidents from the year 2020, and 1388 incidents recorded from the year 2023 we selected those with a quality (resolution and brightness) high enough to allow for the coding and where the conflict was visible from beginning to end. Moreover, in order to detect bystander-bystander interactions, we selected videos which showed at least two bystanders witnessing the conflict and remained on video uninterruptedly for at least 20 s before and after the beginning of the conflict. We followed the same procure in order to be able to detect bystander-antagonists interactions and included in our sample videos where at least one antagonist was visible. In order to meet all the conditions necessary for the analysis, the final sample consisted of 30 clips which involved 131 bystanders, visually identified as 34 women and 97 men.

The conflicts included in the final sample consisted of different types of incidents, including street fights, disputes that resulted from traffic accidents and quarrels occurring outside locals. Conflicts occurred both during the day and in the night-life settings. Although the footage shows the identity of the people recorded, no personal data are known to the researchers, and the coding was based exclusively on the behaviours observed.

### Operational Definition and Coding Procedure

We defined as conflict every interaction between two persons showing at least one aggressive behaviour directed against another person. The aggressive behaviours included can be both physical or non-physical (Table 1; modified from Ejbye-Ernst et al., 2022).

**Table 1:** Behaviours indicating the occurrence of physical or non-physical aggressive interactions (modified from Ejbye-Ernst et al., 2022)

AGGRESSIVE INTERACTION	BEHAVIOURS INCLUDED
Non-physical aggressive interaction	Chasing or charging at someone, invading space, displaying aggressive gestures (e.g., pointing at person, feint, obscene gestures)
Physical aggressive interaction	Kicking, punching, slapping, shoving, head-butting, pushing, throwing or pulling, blocking or holding a person back, wrestling/grappling, hauling a person off, striking with objects

We referred to the people engaging in a conflict as antagonists. The beginning and the end of the conflict were identified with the first and the last aggressive behaviours performed by either antagonist. Bystanders were defined as the individuals included in the frame but not giving or receiving any form of aggressive behaviours to and from the antagonists or actively taking part in the conflict (i.e., showing intervention behaviours included in Ejbye-Ernst, 2022). We restricted our observations to the before-conflict (matched-control, MC) and post-conflict (PC) contexts (Pallante et al., 2024; adaptation from: de Waal & Yoshihara, 1983). The MC context was defined as the period that immediately preceded the moment the bystander noticed the conflict. The MC context ended with the frame preceding the first frame where the bystander noticed the conflict. We considered as “noticing a conflict” every time the bystanders interrupted the ongoing activity they were engaged in and oriented their head and/or body towards the conflict for at least 5 s. When the bystander noticed the conflict, the PC started. Following the coding procedure of Pallante et al. (2023), the observations were conducted only on subjects who were continuously present in the frame with no breaks of more than 10 s. If a bystander moved out of the frame, the coding stopped and was resumed only if the bystander was again in the frame within 10 s. If the bystander was not visible for more than 10 s continuously, we did not resume the observations. This coding strategy allowed us to minimize the risk that bystanders engage in interactions that could potentially affect their emotional state when they were not visible to the coder. Observations were conducted on one bystander for a maximum of 3 min in the PC and for a maximum of 3 min in the MC.

All the behaviours displayed by the bystanders in the PC and in the MC were coded according to a pre-defined ethogram (Table 2). We developed an ethogram which focused on two different categories of behaviours: anxiety-related behaviours and affiliative-related behaviours. The anxiety-related behaviours were modified from the ethogram of distress-related behaviours of Pallante et al. (2024). In particular, we selected from the ethogram of Pallante et al. (2024) only the anxiety-related behaviours, as we aimed at evaluating the variation in the anxiety levels of bystanders. We coded also affiliative behaviours and divided them into physical or non-physical interactions. Physical affiliative behaviours included any form of tactile contact between bystanders or bystanders and antagonists, Non-Physical affiliative behaviours included all forms of interactions or behaviours without any tactile contact between individuals. We coded type and duration of affiliative behaviours (duration ≥2s). We coded the duration of each affiliative behaviours that include a physical contact. In particular, we divided between short affiliative contacts (duration < 10 s) and long affiliative contacts (duration ≥ 10 s). The recording of affiliative behaviours allowed us to divide the PC into two different contexts: PC pre-affiliation and PC post-affiliation. This distinction was made in order to test the effect of the occurrence of affiliation on the expression of anxiety-related behaviours.

**Table 2:** Ethogram of the behaviours considered in the study

**Anxiety Related Behaviours**	
Behavioural Pattern	Definition
Fixing hair – fixh	Setting the hair with a sudden movement of the head (without the use of hands).
Holding the body – holbo	Gripping to the body or parts of it with the arms. It includes: **Holding the back**: placing one or both hands on the posterior part of the body. **Holding the head**: gripping with both hands the head, or placing one hand the forehead, with the index finger and thumb touching the forehead. **Crossing arms**: passing one arm over the other on the chest.
Self-touching – stou	Contacting one’s own body with the hands; the body can be covered by clothes or not. It includes: **Caressing the body**: stroking the body with repeated movements of one or both hands; **Covering the face**: concealing the face or part of it, such as the forehead, the eyes, the nose, the mouth with the palm of the hand(s); **Scratching**: act of rubbing the skin with nails. **Touching body**: making contact with one’s own body with the hands, excluding the face/head zone; **Touching face**: making contact with one’s own face with the hands, it includes biting the nails; **Touching hair**: making contact with one’s own hair with the hands.
Smoking – smo	Inhaling and exhaling smoke by sucking on the end of a lit cigarette, cigar, pipe, etc.
Pacing – pa	Continuous and repetitive walking of an individual remaining located in the same spot, movement in place without a clear direction.
Moving in the place – mop	Motion of the body, especially the legs, while the individual remains located in the same spot, moving the weight from one leg to the other. It includes swinging with the feet, changing the orientation of the body without taking a clear direction, including switch of position in a short timespan (max 2 s apart). Examples: sitting up and standing down repeatedly. Note that the moving in place is usually displayed during the talking with gestures.
Spitting – spit	Expelling liquid from the mouth towards the ground.
Redirected attention – rea	A repetitive action performed with the hands towards an object. It includes repeated displacement of an object from one hand to the other or the regular beating of an object on a surface or on part of the body.
Touching objects – touo	Placing hands into contact with items present on the scene or personal items such as keys, clothes or parts of them (e.g., pockets or a hood of the jacket). The action of touching objects needs to be performed without pursuing the specific purpose of said item (Note: touching objects is not considered when it is a response to a request from someone). It includes: **Arranging**: any action directed with the hands towards the clothes, such as fixing clothes with hands or rolling up the sleeves. **Moving objects**: replacing an item from one hand to the other. it differs from redirected attentions in that it is neither continuous nor repeated. It does not include the movement of the object from one hand to the other when it is followed by an action that does not imply the use of the object (i.e., when it is performed to free one hand from the object to achieve a specific purpose, such as grabbing something else).
**Affliliative behaviours without physical contact**	
Behavioural Pattern	Definition
Proximity – prox	Standing close to another person at a distance of maximum 1m.
Interacting Non-Physical – intnp	Engaging in verbal communication without any physical touch directed towards the individual involved in the interaction.
**Affliliative behaviours with physical contact**	
Behavioural Pattern	Definition
Interacting Physical Affiliative – intpa	Engaging in verbal communication where physical touch is directed towards the individual involved in the interaction. Physical touch includes brief touch of the body of another person in a friendly or peaceful manner (for example: touching the shoulder, stroking the back, hugging).
Close contact – contactc	Standing/walking next to another bystander with physical contact for at least 2 seconds.
Touch – touch	Physically contacting another individual with one or two hands; brief contact no longer than 2 seconds. It occurs outside a verbal interaction.

After a training period of one month, we checked the reliability of the ethogram with a second independent coder by double-coding and comparing 10% of the videos via Cohen’s Kappa (κ), and we obtained an interobserver reliability score of 0.89 (almost perfect agreement).

### Statistical Analysis

To check whether the occurrence of the conflict affected the probability of observing the behaviour of interest, we compared the presence/absence of the behaviours in the PC/MC conditions by running Generalized Linear Mixed Models (GLMMs). In particular, models were run for anxiety-related behaviours (GLMM_1_, N_occurrence_=376 records), affiliative behaviours with no contact (GLMM_2_, *N* = 374 records), affiliative behaviours with all types of contact (GLMM_3_, N_occurrence_=441 records), affiliative behaviours with short contact (GLMM_4_, N_occurrence_=359 records) and affiliative behaviours with long contact (GLMM_5_, N_occurrence_=441 records). In all models, we entered the occurrence of the behaviour of interest as target, binary variable (absence/presence) and the condition as the fixed factor (binary, PC/MC). To test the variation in the occurrence of anxiety-related behaviours in GLMM_1_, we included the occurrence of anxiety-related behaviours only before or in absence of the occurrence of any affiliative behaviour with contact. Similarly, to test the variation in the occurrence of affiliative behaviours with no contact in GLMM_2_, we included the occurrence of affiliative behaviours with no contact only before or in absence of the occurrence of any affiliative behaviour with contact. Finally, to test the variation in the occurrence of affiliative behaviours with short contact in GLMM_4_, we included the occurrence of affiliative behaviours with short contact only before or in absence of the occurrence of any affiliative behaviour with long contact. For all models, the identity of the bystanders was entered as a random factor, as alphanumeric code.

We ran a second set of GLMMs on the affiliative behaviours on which the condtion had a significant main effect in order to test whether bystanders preferentially directed them towards the antagonists (triadic interactions) or towards the other bystanders (quadratic interactions). First, we ran models for long physical affiliative behaviours (GLMM_6_, N_occurrence_= 71 records), and non-physical affiliative behaviours (GLMM_7,_ N_occurrence_= 332 records). In these models, we entered as dependent, binary variable the occurrence of the behaviour in PC/MC conditions (absence = 0; presence = 1). We included as fixed factor the type of interaction displayed by the bystanders (numeric, both triadic and quadratic interactions = 2, triadic interaction = 3, quadratic interaction = 4). Second, we restricted the analysis to the first affiliative contact to check whether bystanders preferentially contacted antagonists or bystanders. We ran models for long physical affiliative behaviours (GLMM_8_, N_occurrence_= 43 records), and non-physical affiliative behaviours (GLMM_9,_ N_occurrence_= 200 records). We entered as dependent, binary variable the occurrence of the behaviour in the PC (absence = 0; presence = 1) and as fixed factor the type of interaction displayed by the bystanders (binomial, triadic interaction = 3, quadratic interaction = 4). The identity of the bystanders was entered as a random factor, as alphanumeric code, in all models.

We run the analysis with R 4.1.3 version. The models were fitted in R [(R Core Team, 2019); version 3.5.3] by using the function glmer of the R-package lme4 (Bates et al., 2015). As a first step we verified if the full model significantly differed from the null model, including only the random factors (Forstmeier & Schielzeth, 2011). The likelihood ratio test (Dobson, 2002) was used to test this significance (ANOVA with argument “Chisq”). Subsequently, by using the R-function “drop1,” the p-values for the individual predictors based on likelihood ratio tests between the full and the null model were calculated (Barr et al., 2013). As the response variable was binary, a binomial error distribution was used (link function: logit).

In order to test for the variation in the occurrence of anxiety-related behaviours between contexts, we ran a Kruskal Wallis test. In particular, we compared the frequency of anxiety-related behaviours (total count of anxiety-related behaviours/total time of observation per subject) in two different conditions: PC pre-affiliation and PC post-affiliation. We tested only the affiliative behaviours that resulted significant in the PC/MC models, i.e., non-physical affiliation and long physical affiliation, by running two separated tests. We selected bystanders with at least 10 s of observation in each condition.

## Results

### Anxiety-Related and Affiliative Behaviour According to Contexts (PC/MC)

**Fig. 1:**
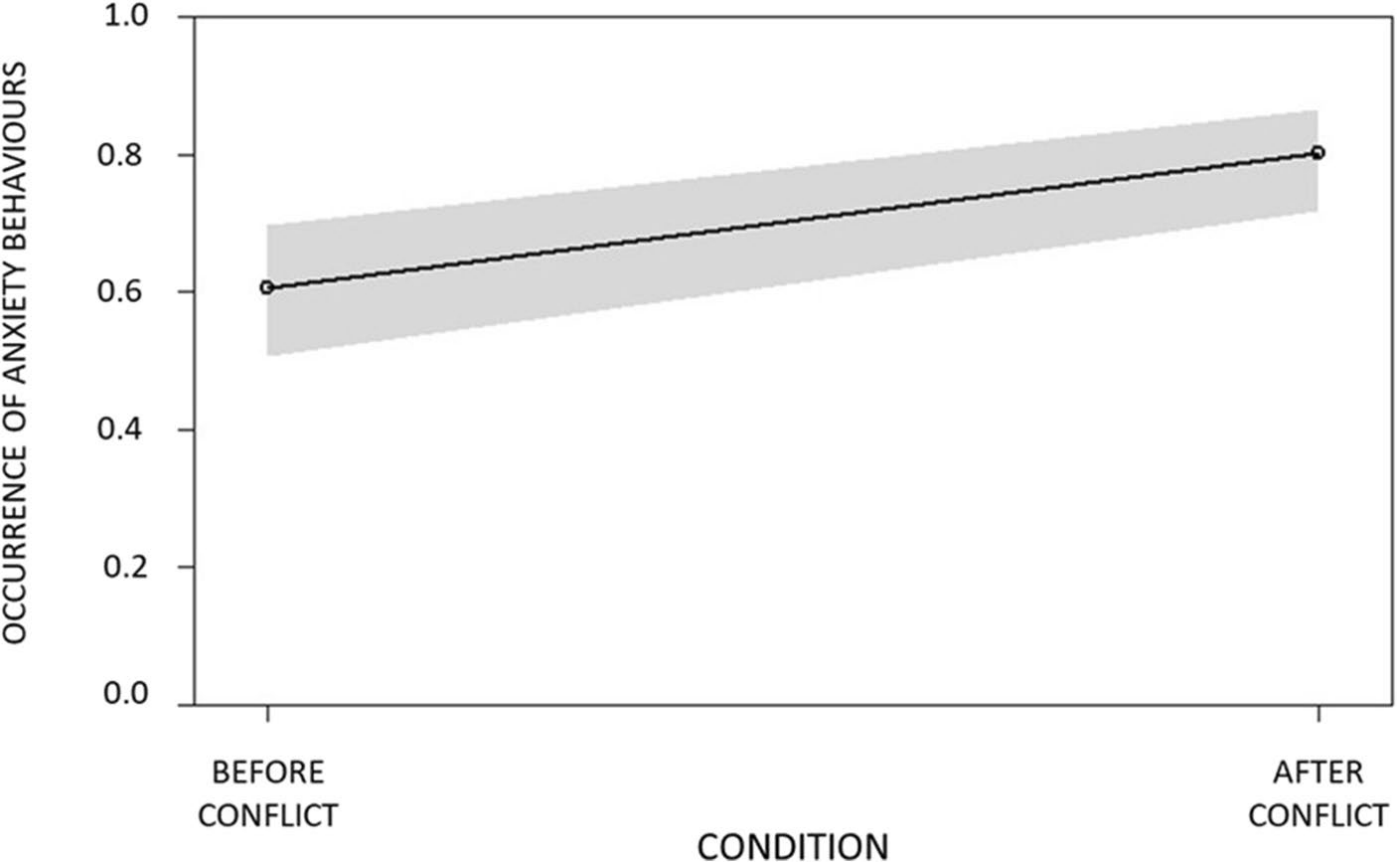
Variation in occurrence of anxiety behaviours in the post-conflict (PC) compared with the matched-control (MC) condition. Error margins refer to 95% confidence limits

**Fig. 2:**
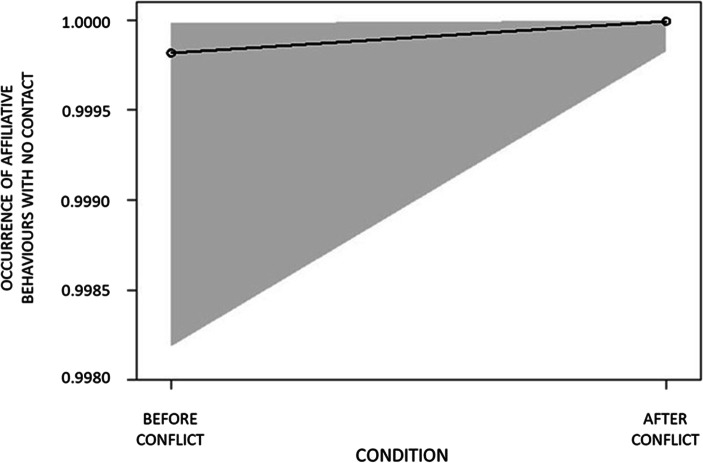
Variation in occurrence of affiliative behaviours with no contact in the post-conflict (PC) compared with the matched-control (MC) condition

**Fig. 3:**
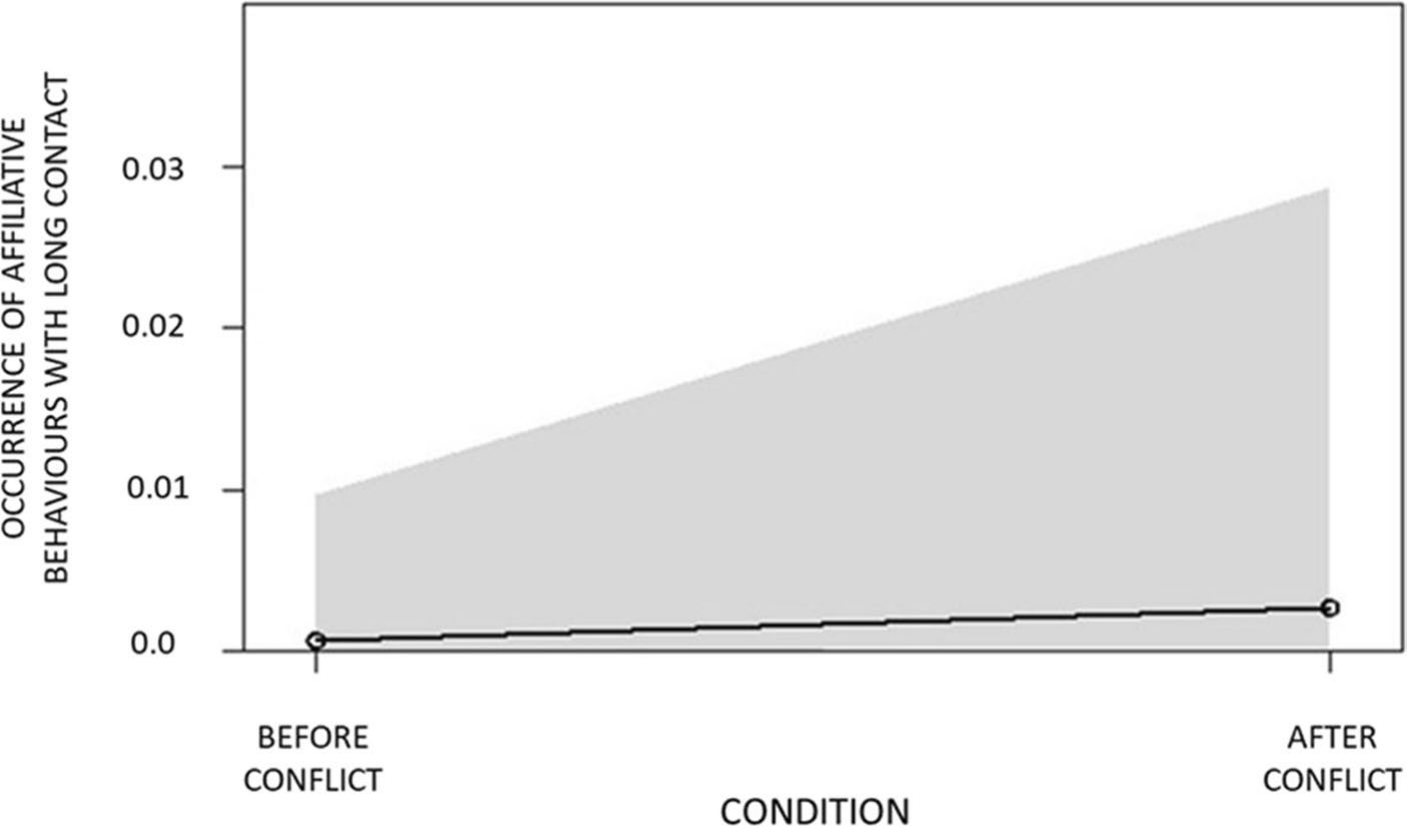
Variation in occurrence of affiliative behaviours with long contact in the post-conflict (PC) compared with the matched-control (MC) condition. Error margins refer to 95% confidence limits

In GLMM_1_ (anxiety-related behaviour), GLMM_2_ (affiliative behaviours with no contact) and GLMM_5_ (affiliative behaviours with long contact), the full model including condition (PC/MC) as a fixed factor differed significantly from the null model including the random factor (Table 3). Hence, the variance explained by the test predictors was significantly different from the variance explained by the variables in the null model. Therefore, we proceeded with the drop 1 procedure to check for the effect of the individual predictor. We found that condition (PC/MC) had a significant effect on the presence of anxiety-related behaviour (Table 3; Fig. 1), affiliative behaviours with no contact (Table 3; Fig. 2) and affiliative behaviours with long contact (Table 3; Fig. 3). In particular, all these behaviours were more frequent in PC than in MC.

**Table 3:** Full results of the influence of the condition (PC/MC) on the relative occurrence of anxiety behaviour (GLMM_1_); non-physical affiliative behaviour (GLMM_2_); all types physical affiliative behaviours (GLMM_3_); short physical affiliative behaviours (GLMM_4_); long physical affiliative behaviours (GLMM_5_). For all models the bystander’s identity (bystander ID) was included as random factor

Predictors	Estimates	SEM	CI_95_	Effect size	ꭓ^2^	*P*
GLMM_1_ anxiety behaviour	Full vs. null model: χ^2^ = 13.833, df = 1, *p* < 0.001					
(Intercept)	0.434	0.207	a	a	a	a
Condition (PC)^b^	0.964	0.266	0.442, 1.486	0.802	0.623	**0.001**
GLMM_2_ non-physical affiliative behaviour	Full vs. null model: χ^2^ = 18.929, df = 1, *p* < 0.001					
(Intercept)	8.604	1.169	a	a	a	a
Condition (PC)^b^	3.525	1.067	1.432, 5.617	0.999	3.302	**0.001**
GLMM_3_ all type physical affiliative behaviour	Full vs. null model: χ^2^ = 0.778, df = 1, *p* = 0.378					
(Intercept)	−1.593	0.308	a	a	a	a
Condition (PC)^b^	0.239	0.273	−0.295, 0.773	0.205	0.878	0.380
GLMM_4_ short physical affiliative behaviour	Full vs. null model: χ^2^ = 0.887, df = 1, *p* = 0.346					
(Intercept)	−1.983	0.328	a	a	a	a
Condition (PC)^b^	−0.309	0.328	−0.951, 0.334	0.091	−0.941	0.347
GLMM_5_ long physical affiliative behaviour	Full vs. null model: χ^2^ = 10.305, df = 1, *p* = 0.001					
(Intercept)	−7.405	1.416	a	a	a	a
Condition (PC)^b^	1.509	0.513	0.504, 2.513	0.003	2.943	**0.003**

^a^Not shown as not having a meaningful interpretation

^b^ These predictors were dummy-coded, with the reference category as MC

For GLMM_3_ (affiliative behaviours with all types of contact) and GLMM_4_ (affiliative behaviours with short contact), we found no significant difference between the full and null models (Table 3); hence, the condition (PC/MC) exerted no effect on these types of affiliative behaviours, and we did not proceed with the drop 1 procedure.

### Triadic and Quadratic Affiliation

We found that bystanders did not preferentially direct long physical affiliative behaviours (GLMM_6_, target variable: presence/absence of long-physical affiliative behaviour) and non-physical affiliative behaviour (GLMM_7_, target variable: presence/absence of non-physical affiliative behaviour) towards antagonists (triadic interaction) or towards other bystanders (quadratic interaction; see Table S1 for the results). Similarly, when restricting the analysis to the first affiliative contact, we did not find any preference to direct long physical affiliative behaviours (GLMM_8_ target variable: presence/absence of long-physical affiliative behaviour) and non-physical affiliative behaviour (GLMM_9_, target variable: presence/absence of non-physical affiliative behaviour) towards antagonists (triadic interaction) or towards other bystanders (quadratic interaction; see Table S2 for the results).

### Anxiety-Related Behaviours Before and After Post-Conflict Affiliation

**Fig. 4:**
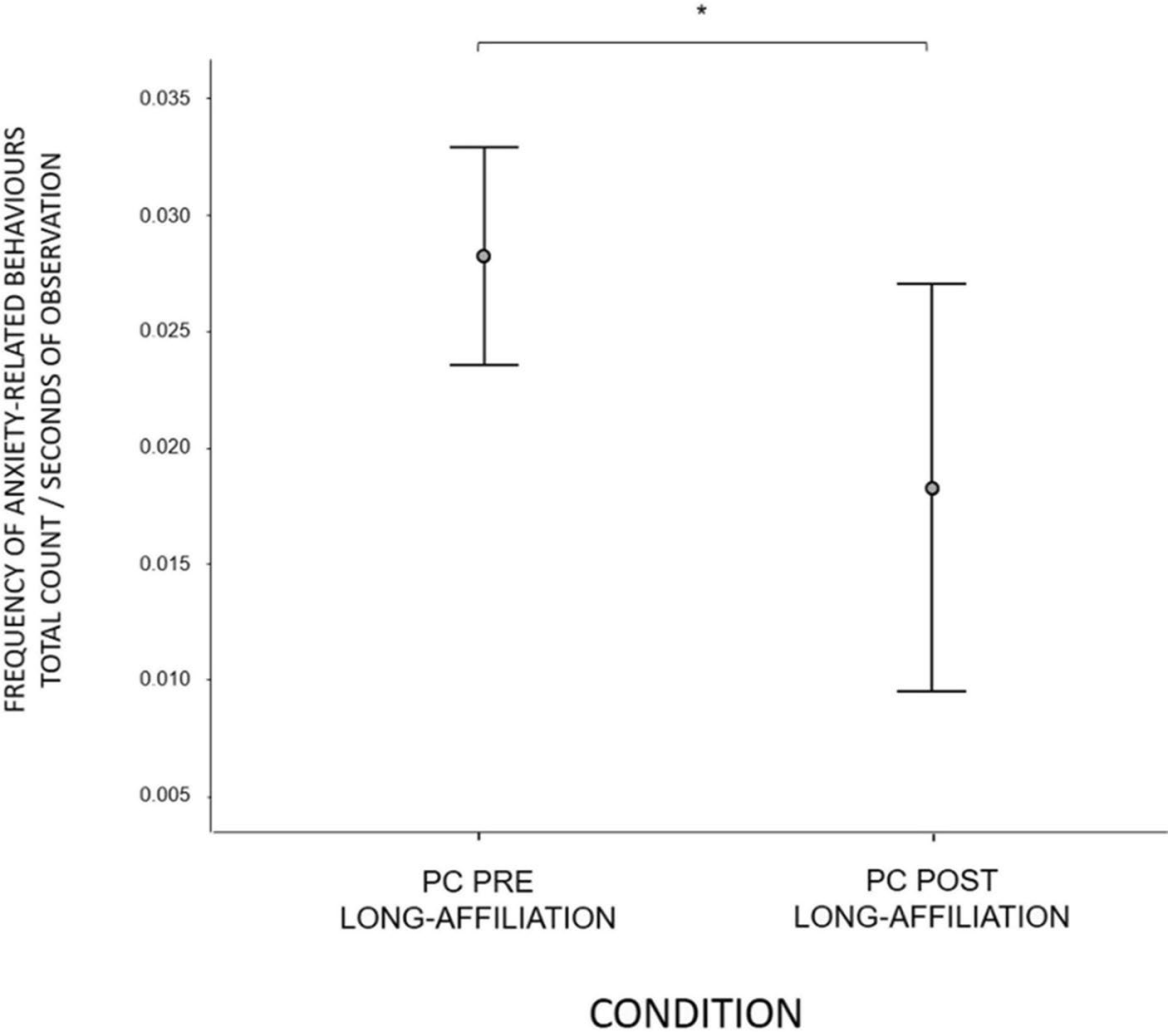
Comparison of the occurrence of anxiety-related behaviours in the conditions PC pre long-affiliation and PC post long-affiliation. Asterisks indicate significance

Finally, we compared the frequency of anxiety-related behaviours in two conditions: PC before affiliation and PC post affiliation. We found that bystanders significantly decreased the frequency of anxiety-related behaviours after the occurrence of long affiliation (PC before long affiliation vs. PC post long affiliation, *H*(1) = 5.255, *N* = 148, *p* = 0.022; Fig. 4). However, no difference in the frequency of anxiety was found before and after non-physical affiliation PC before non-physical affiliation vs. PC post non-physical affiliation, *H*(1) = 0.849, *N* = 136, *p* = 0.357).

## Discussion

In this paper, we investigated the relationship between post-conflict affiliation and bystanders’ anxiety. We found that the occurrence of a street fight increased the expression of anxiety-related behaviours in bystanders, and it influenced the probability that bystanders became involved in nonphysical affiliation, such as proximity and verbal interaction, and long-lasting physical affiliation, such as staying in physical contact with a person. We did not find an increase in short physical affiliation behaviours, such as physically touching a person with a brief touch. Long lasting affiliation reduced, in turn, the occurrence of anxiety-related behaviours. In all models, the effect size ranged from negligible to large, pointing at the possible effect of different variables that might have played a role in explaining the increase of anxiety and affiliation, and that we did not take into account in the present study.

The observed increased expression of anxiety-related behaviours reveals that not only antagonists (Pallante et al., 2024), but also bystanders show such tendency in the post-conflict context. By extending it to bystanders, this finding suggests that conflicts generate social tension at group level – not just for the people directly involved as antagonists in the conflict – as is also the case in nonhuman primates (Cordoni et al., 2025; Daniel & Alves, 2015; De Marco et al., 2010; Judge & Mullen, 2005). Such stressful experience can have, in turn, a positive effect on the bystanders’ propensity to affiliate and interact. Consistently, we found in fact that social affiliation increases in the post-conflict context. The increase of social affiliation of bystanders can be interpreted in two ways. The affiliative interaction can be a tension-reduction mechanism that bystanders adopt to cope with the social tension generated by the conflict. Affiliation would have, therefore, a buffer effect on the emotional state of the bystanders, as documented in studies on human behaviour in stressful situations (Grandi, 2016; Kikusui et al., 2006; Morrison, 2016). This result is in line with observations of nonhuman primates, where bystanders of conflict events affiliate with each other to reduce their arousal (Daniel & Alves, 2015; Judge & Mullen, 2005; Schino & Sciarretta, 2015). Moreover, it has been predicted that affiliating with antagonists can also have a positive effect on the emotional state of bystanders (Decety et al., 2016; de Waal & Preston, 2017).Although contacting the conflict parties may reflect the bystanders’ prosocial tendencies (Clay & de Waal, 2013; de Waal & Preston, 2017; Fraser et al., 2008; Norscia & Palagi, 2013; Romero et al., 2010), it can also improve bystanders’ emotional state. Similar dynamics have also been observed in nonhuman primates and social-living mammals, so the basic mechanisms underlying them may be rooted in mammalian evolutionary history (Cordoni et al., 2023; Fraser et al., 2008; Palagi et al., 2014).

The social tension generated by the conflict and the subsequent increase of affiliation as a coping mechanism has frequently been interpreted as a social consequence of living in groups (de Waal, 2000). Conflicts in nonhuman primates are, as a matter of fact, social phenomena, as they can have consequences on the network of relationships between group members (de Waal, 2000). Affiliation as a buffer mechanism to social tension has evolved to prevent the disruption of the group, and has, therefore, an adaptive function (de Waal, 2008). The increase of social tension and the following consequent affiliation as a coping strategy in human street fights, calls for reflections on how we define human groups. Street fights occur in public places, partly characterized by a blend of people who might be stranger to each other, and as such only partly form what we would usually consider a group. Nonetheless, such heterogeneity in group composition is representative of human societies. In contrast, in nonhuman primates most aggression and post-conflict affiliation occur within cohesive social networks characterized by enduring social relationships, often among related or long-term associated individuals, where social bonds and kinship play a central role in conflict management and tension-reduction processes, affecting the likelihood of bystanders’ intervention and emotional state (e.g., Fraser et al., 2008; Judge & Mullen, 2005; Romero et al., 2010). Our observations suggest that the differences in group structure in human and nonhuman primates do not prevent the occurrence of similar conflict management strategies, which have been conserved over time as they favour group cohesion, a common feature of all social animals, including humans. In addition, by interacting in tense situations, people might develop “interactional relationships” that guide affiliative behaviours during conflicts (Lindegaard & Liebst, 2025). In conclusion, with due considerations on species-specificities, naturalistic observations on human behaviour in public spaces allow for an increased comparability with other nonhuman primates species.

Our observation show that only long physical affiliation is followed by a reduction of anxiety related behaviours in the bystanders. Although in nonhuman primates brief physical contacts are usually exchanged with an appeasement function in the post-conflict context, only long-lasting physical affiliation has been associated with a pleasant, calming effect in the receiver (Aureli & Schino, 2022). In humans, this effect is generated not only by continuous contact, but also by the gentle stroke of skin (Grandi, 2016; McGlone et al., 2007). In our coding, we accounted for both types of affiliation, as we categorized as long-lasting physical affiliation every interaction with continuous or repeated physical contact lasting more than 10 s. Our results suggest, therefore, that a brief, isolated, touch might not be perceived as affective as a prolonged physical contact and play an effective role in reducing anxiety.

A possible limitation of the current study concerns the low variability of our data. The footage analysed were recorded exclusively in Amsterdam, thus restricting our sample to a specific location. Considering footage from different cities and national contexts would increase the generalizability of our results. Moreover, the footage analysed in this study lacked sound. Therefore, we were not able to detect whether affiliation could have occurred through more subtle verbal interactions that were not visible through the behaviours observed, nor to consider the content of the conversation, which might have an impact on bystanders’ emotional state. We hope that this issue will be buffered by future technology that might provide CCV footage including sounds and we encourage future studies to consider verbal characteristics as potential factors affecting bystanders’ social dynamics. Despite this limitation, the data used in this study drew from a large sample and consider humans in their naturally occurring conflictual interactions without space constraints or without being uprooted from the social context where the interactions occur. Video footage represents, therefore, a unique source that allows to conduct naturalistic observations on humans’ spontaneous behaviours, offering the opportunity to disclose differences and similarities through a cross-species perspective.

This study introduced for the first time the role of the emotional state of bystanders on their social predispositions in conflict situations. By drawing from the ethological method, it allowed to highlight that, similarly to what has been documented in nonhuman primates, the eruption of a conflict increases social tension and social tendencies in bystanders. We suggest that the adoption of a behavioural-based methodology offers the unique opportunity to investigate the role of the emotional state as a factor explaining bystander activity during conflicts. Moreover, by allowing for a cross-species comparison, it introduces biological explanations to the evolution of human social behaviour and provides empirical evidence of the models explaining prosocial behaviours developed and applied, until now, exclusively on nonhuman primates. We propose that the integration of biological and sociological perspectives is key to provide a comprehensive picture of bystanders’ social dynamics in conflict context.

## Supplementary Information

Below is the link to the electronic supplementary material.

Supplementary Material 1 (DOCX 24.0 KB)

Supplementary Material 2 (DOCX 24. 0 KB)

Supplementary Material 3 (ZIP 21.2 KB)

## Data Availability

The data associated with this research are available as supplementary material.

## Declarations

The study was authorized by the Netherlands Public Prosecution Service and the National Police Force and approved by the Ethics Advisory Board at the Amsterdam Institute for Social Science Research, the University of Amsterdam (2022-AISSR-15534).
