# Supplementary material for: Affiliation as a Social Tension Buffer in the Aftermath of Street Fights
Source: Hum Nat. 2026 Mar 17;37(1):58–76. doi: 10.1007/s12110-026-09516-1 (PMC13079545; doi:10.1007/s12110-026-09516-1)
Supplement: Supplementary file 1 [file 12110_2026_9516_MOESM1_ESM.docx]

Affiliation as a social tension buffer in the aftermath of street fights

Human Nature

Laura Pighinia,b, Ivan Norsciac*, Marie Rosenkrantz Lindegaardb, Virginia Pallantea*

a Netherlands Institute for the Study of Crime and Law Enforcement (NSCR), Amsterdam, The Netherlands

b Department of Sociology, VU University Amsterdam, Amsterdam, The Netherlands

c Department of Life Sciences and Systems Biology, University of Turin, Turin, Italy

*Corresponding authors: VPallante@nscr.nl, ivan.norscia@unito.it

Table S2 Full results of GLMM8 (long physical affiliative behaviours) on the first type of interaction influencing the occurrence of the presence of long physical affiliative behaviours and GLMM9 (non-physical affiliative behaviour) on the first type of interaction influencing the occurrence of the presence of non-physical affiliative behaviour. For all models the bystander’s identity (bystander ID) was included as random factor

a Not shown as not having a meaningful interpretation

b These predictors were dummy-coded, with the reference category: triadic interaction

| Predictors | Estimates | SEM | CI95 | Effect size | ꭓ2 | P |
| --- | --- | --- | --- | --- | --- | --- |
| GLMM8 long physical affiliative behaviour | Full vs. null model:   χ2=0.001, df=1, p=0.978 |
| (Intercept) | 1.099 | 1.155 | a | a | a | a |
| Type of interaction (quadratic)b | 0.034 | 1.212 | -2.409, 2.341 | -0.034 | -0.028 | 0.978 |
| GLMM9 non-physical affiliative behaviour | Full vs. null model:   χ2=2.629, df=1, p=0.105 |
| (Intercept) | 0.588 | 0.558 | a | a | a | a |
| Type of interaction (quadratic)b | -0.913 | 0.577 | -2.044, 0.218 | -0.913 | -1.582 | 0.114 |
