# Supplementary material for: Affiliation as a Social Tension Buffer in the Aftermath of Street Fights
Source: Hum Nat. 2026 Mar 17;37(1):58–76. doi: 10.1007/s12110-026-09516-1 (PMC13079545; doi:10.1007/s12110-026-09516-1)
Supplement: Supplementary file 2 [file 12110_2026_9516_MOESM2_ESM.docx]

Affiliation as a social tension buffer in the aftermath of street fights

Human Nature

Laura Pighinia,b, Ivan Norsciac*, Marie Rosenkrantz Lindegaardb, Virginia Pallantea*

a Netherlands Institute for the Study of Crime and Law Enforcement (NSCR), Amsterdam, The Netherlands

b Department of Sociology, VU University Amsterdam, Amsterdam, The Netherlands

c Department of Life Sciences and Systems Biology, University of Turin, Turin, Italy

*Corresponding authors: VPallante@nscr.nl, ivan.norscia@unito.it

Table S1 Full results of GLMM6 (long physical affiliative behaviours) on the type of interaction influencing the occurrence of the presence of long physical affiliative behaviours and GLMM7 (non-physical affiliative behaviour) on the type of interaction influencing the occurrence of the presence of non-physical affiliative behaviour. For all models the bystander’s identity (bystander ID) was included as random factor

a Not shown as not having a meaningful interpretation

b These predictors were dummy-coded, with the reference category: both triadic and quadratic interactions

| Predictors | Estimates | SEM | CI95 | Effect size | ꭓ2 | P |
| --- | --- | --- | --- | --- | --- | --- |
| GLMM6 long physical affiliative behaviour | Full vs. null model:   χ2=1.536, df=2, p=0.464 |
| (Intercept) | 17.494 | 724.078 | a | a | a | a |
| Type of interaction (triadic)b | -16.777 | 724.077 | -1435.943, 1402.388 | -16.777 | -0.023 | 0.982 |
| Type of interaction (quadratic)b | -16.761 | 724.078 | -1435.927, 1402.406 | -16.761 | -0.023 | 0.982 |
| GLMM7 non-physical affiliative behaviour | Full vs. null model:   χ2=4.843, df=2, p=0.089 |
| (Intercept) | 0.511 | 0.327 | a | a | a | a |
| Type of interaction (triadic)b | -0.223 | 0.831 | -1.851, 1.405 | -0.223 | -0.269 | 0.788 |
| Type of interaction (quadratic)b | -0.729 | 0.348 | -1.411, -0.048 | -0.729 | -2.098 | 0.036 |
